# Lactobacillus rhamnosus GG and butyrate supplementation in rats with bone cancer reduces mechanical allodynia and increases expression of μ-opioid receptor in the spinal cord

**DOI:** 10.3389/fnmol.2023.1207911

**Published:** 2023-06-14

**Authors:** Wenxi Yuan, Jie Xiao, Huabao Liao, Zhiyuan Xie, Yiran Zhao, Cheng Li, Keying Zhou, Xue-Jun Song

**Affiliations:** ^1^Department of Medical Neuroscience, School of Medicine, Southern University of Science and Technology, Shenzhen, China; ^2^SUSTech Center for Pain Medicine, School of Medicine, Southern University of Science and Technology, Shenzhen, China; ^3^Department of Pediatrics, The First Affiliated Hospital, Southern University of Science and Technology, Shenzhen, China

**Keywords:** Lactobacillus rhamnosus GG, cancer pain, mechanical allodynia, μ-opioid receptor, gut microbiota, butyrate, histone deacetylase 2

## Abstract

**Introduction:**

Chronic cancer pain is one of the most unbearable symptoms for the patients with advanced cancer. The treatment of cancer pain continues to possess a major challenge. Here, we report that adjusting gut microbiota via probiotics can reduce bone cancer pain (BCP) in rats.

**Methods:**

The model of BCP was produced by tumor cell implantation (TCI) to the tibia in rats. Continuous feeding of Lactobacillus rhamnosus GG (LGG) was used to modulate the gut microbiota. Mechanical allodynia, bone destruction, fecal microbiota, and neurochemical changes in the primary dorsal root ganglion (DRG) and the spinal dorsal horn (DH) were assessed.

**Results:**

LGG supplementation (10^9^ CFU/rat/day) delayed the production of BCP for 3–4 days and significantly alleviated mechanical allodynia within the first 2 weeks after TCI. TCI-induced proinflammatory cytokines TNF-α and IL-β in the DH, and TCI-induced bone destruction in the tibia were both significantly reduced following LGG supplementation examined on day 8 after TCI. Meanwhile, we found that LGG supplementation, in addition to inhibiting TCI-induced pain, resulted in a significantly increased expression of the μ-opioid receptor (MOR) in the DH, but not in the DRG. LGG supplementation significantly potentiated the analgesic effect of morphine. Furthermore, LGG supplementation led to an increase in butyrate levels in the feces and serum and a decrease in histone deacetylase 2 (HDAC2) expression in the DH. Feeding TCI-rats with sodium butyrate solution alone, at a dose of 100 mg/kg, resulted in decreased pain, as well as decreased HDAC2 expression and increased MOR expression in the DH. The increased expression of MOR and decreased HDAC2 were also observed in neuro-2a cells when we treated the cells with serum from TCI rats with supplementation of LGG or sodium butyrate.

**Discussion:**

This study provides evidence that reshaping the gut microbiota with probiotics LGG can delay the onset of cancer pain. The butyrate-HDAC2-MOR pathway may be the underlying mechanism for the analgesic effect of LGG. These findings shed light on an effective, safe, and non-invasive approach for cancer pain control and support the clinical implication of probiotics supplementation for patients with BCP.

## 1. Introduction

The gut microbiota constitutes the most intricate and abundant micro-ecological system within the human body, and the homeostasis between microbiota and host in the gastrointestinal (GI) tract is critical for healthy maintenance (Guo et al., 2019). The microbiota-gut-brain axis describes signaling and communication between microbiota, gut, and brain. As an essential part of this axis, the gut microbiota has been found involved in the pathogenesis of central nervous system (CNS) diseases like autism, depression, anxiety, and some pain disorders (Ma et al., 2022). Though studies have explored the relationship between gut microbiota and chronic pain (Amaral et al., 2008; Shen et al., 2017; Ma et al., 2022), it remains unclear whether modulating gut microbiota could affect the development of bone cancer pain (BCP).

BCP, one of the most unbearable symptoms presented in patients with advanced cancer, exhibits some characteristics similar to inflammatory and neuropathic pain (Liu et al., 2013; Zajączkowska et al., 2019) and is usually accompanied with central inflammation and bone destruction (Liu et al., 2013; Mantyh, 2013). The changed gut microbiome and impaired intestinal barrier function were also found in patients with cancer pain compared to cancer patients without pain (Zhang et al., 2022). The μ-opioid receptor (MOR) is a G-protein coupled receptor (GPCR) that suppresses neuron excitability through inhibition of cyclic AMP, voltage-gated calcium channel (VGCC), and G protein inwardly rectifying potassium (GIRK) when activated by agonists (Corder et al., 2018). Studies have reported a reduction in the expression of MOR in the dorsal root ganglia (DRG) and the spinal dorsal horn (DH) in mice with BCP (Yamamoto et al., 2008; Zhu et al., 2017). Restoration of expression of MOR in the spinal cord by histone deacetylase (HDAC) inhibitor could alleviate mechanical allodynia (Hou et al., 2017). Studies have also reported the possible epigenetic regulation of MOR by HDAC (Kim et al., 2004; Hwang et al., 2007; Hou et al., 2017). However, it remains unclear whether modulating gut microbiota could have an impact on MOR in the nervous system that is directly related to analgesia.

Probiotic Lactobacillus rhamnosus GG (LGG) exhibits multiple health benefits like antitumor (Vivarelli et al., 2019; Owens et al., 2021), immune system stimulation, bone formation (Tyagi et al., 2018), and alleviating abdominal pain (Horvath et al., 2011; Trivić et al., 2021). LGG was found to boost butyrate synthesis in the GI tract of mice (Tyagi et al., 2018). As one of the most beneficial short-chain fatty acids (SCFAs), butyrate is an inhibitor of HDAC (Arpaia et al., 2013; Guo et al., 2019) and can suppress neuropathic pain (Kukkar et al., 2014) and visceral pain (Russo et al., 2016). However, whether and how LGG and butyrate alleviate BCP remains unknown. In this study, we examined the effects of oral administration of LGG and combination therapy of LGG with morphine on BCP in rats and investigated the underlying mechanisms through the metabolite pathway. We found that LGG supplementation can alleviate BCP and potentiate analgesic effect of morphine, which potentially attributed to the increased expression of MOR in the DH via elevated level of butyrate in serum through HDAC2 inhibition. This study indicates that LGG and butyrate may be used in clinic as an effective approach for alleviating BCP.

## 2. Materials and methods

### 2.1. Animals, drugs, and drug administration

Adult, female SPF Sprague-Dawley (SD) rats (6–7 weeks with 180–200 g-wt; and about 4 weeks with 70–80 g-wt) were purchased from the Experimental Animal Center, Southern University of Science and Technology (SUSTech). Animals were housed in pairs at the animal center and maintained under a normal 12 h light:12 h dark schedule, with *ad libitum* access to food and water. All protocols were approved by the Animal Care and Use Committee of SUSTech. All surgical procedures were performed under anesthesia with 2% isoflurane (RWD, R510-22-16, Shenzhen, China).

**FIGURE 1:**
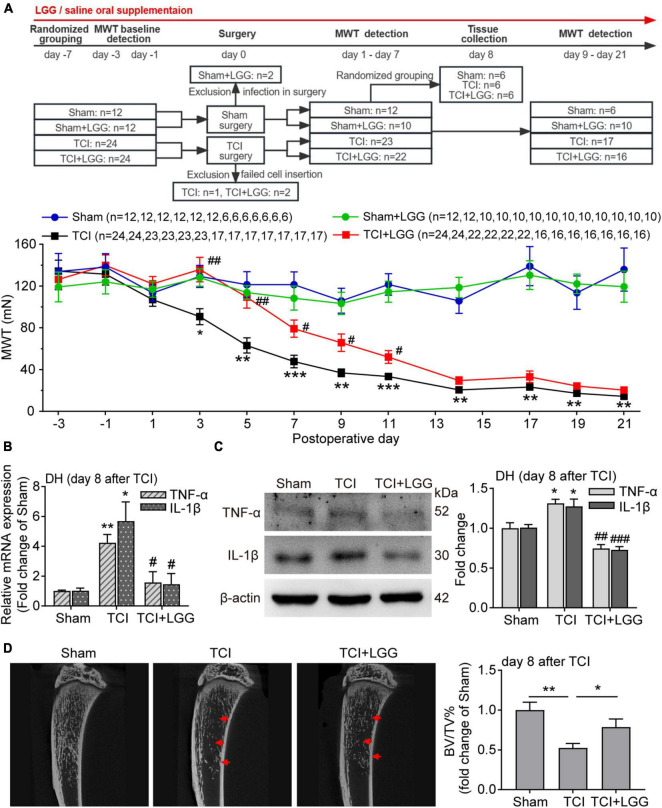
LGG supplementation alleviates TCI-induced mechanical allodynia, spinal inflammation and the bone destruction in rats. **(A)** Reduced mechanical allodynia manifested as increased MWT following the oral supplementation of LGG (1 × 10^9^ CFU/day) in TCI-rats. Two-way ANOVA with Tukey’s multiple comparison test, *p < 0.05, **p < 0.01, ***p < 0.001 TCI vs. Sham; #*p* < 0.05, ##*p* < 0.01 TCI + LGG vs. TCI. LGG, Lactobacillus rhamnosus GG; MWT, mechanical withdrawal threshold; TCI, tumor cell implantation. **(B,C)** Expression of tumor necrosis factor alpha (TNF-α) and interleukin-1beta (IL-1β) mRNA **(B)** and protein **(C)** in the spinal DH from rats on day 8 after TCI. *n* = 3 in each group. One-way ANOVA with Dunnett’s multiple comparison test, *p < 0.05, **p < 0.01 TCI vs. Sham; #*p* < 0.05, ##*p* < 0.01, ###*p* < 0.001 TCI + LGG vs. TCI. DH, dorsal horn. **(D)** Representative micro-CT images (left) showing bone microstructure, and quantification (right) showing BV/TV in the proximal part of tibia trabecular bone from rats on day 8 after TCI. *n* = 4 in Sham, *n* = 6 in other groups. One-way ANOVA with Dunnett’s multiple comparison test, *p < 0.05, **p < 0.01. BV/TV, bone volume/tissue volume.

For probiotic treatment, LGG (ATCC53103) was supplied at a dosage of 1 × 10^9^ CFU/day in 1 ml sterilized saline by oral gavage (Tyagi et al., 2018), which started one week prior to tumor cell implantation (TCI) surgery for stabilizing colonization of LGG in the GI tract, and continued for up to 3 weeks after surgery or till sacrifice on day 8 (Figure 1A). For butyrate treatment, sodium butyrate (Coolaber, CS9931, Beijing, China) was supplied at a dosage of 100 mg/kg/day in 1 ml sterilized saline by oral gavage (Kukkar et al., 2014; Russo et al., 2016), which started 0.5 h before TCI surgery (day 0) and continued for up to 3 weeks after surgery or till sacrifice on day 8. The rats from groups of Sham and TCI received 1 ml of sterilized saline in the same protocol. Hydrochloride morphine was injected intrathecally at a dosage of 0.1, 1, or 10 μg in 10 μl sterilized saline by means of lumbar puncture at the intervertebral space of L4–5 under a brief light isoflurane anesthesia.

### 2.2. Animal models of BCP

Walker-256 mammary gland carcinoma cells (1 × 10^7^ in 0.5 ml sterile saline) were injected intraperitoneally (i.p.) into female rats (70∼80 g). One week after injection, the tumor cells were extracted from the ascitic fluid, which were centrifuged to get the cell pallet and resuspended with cold sterile saline to the concentration of 1 × 10^5^ cells/μl. The extracted tumor cells (5 μl) were used to inject into the intramedullary space of the left tibia in naïve rats (180–200 g) to produce bone cancer and BCP. The protocols were similar to that previously described (Liu et al., 2013; Zhai et al., 2021). In brief, the rats were lightly anesthetized with isoflurane, and a superficial incision, approximately 1 cm in length, was made proximal to the knee to expose the left tibia. A 23-gauge needle which contains a tumor cell “sandwich” with 5 μl air, 5 μl suspension of tumor cells (5 × 10^5^), and 5 μl air was inserted into the tibia cavity. The contents of the syringe were slowly injected, and the needle was kept in place for 2 min after injection to allow for cell diffusion. The needle hole was sealed with bone wax upon needle withdrawal, and the bone surface of the injection site was sealed with dental bone cement to prevent the leakage of tumor cells. Boiled tumor cells were injected as sham control.

### 2.3. Assessment of mechanical allodynia

In this study, mechanical allodynia was selected as the indicator of BCP. Mechanical allodynia was evaluated by measuring the mechanical withdrawal threshold (MWT), which was tested with von Frey filaments (Aesthesio, Ugo, Italy). The 50% paw withdrawal threshold was determined by the up-down method as described previously (Chaplan et al., 1994; Zhai et al., 2021). In brief, the animals were placed individually in a box on an elevated wire grid for acclimation for 30 min. The von Frey filaments ranging from 2 to 60 g were applied perpendicularly to the mid-plantar surface of the left hind paw (ipsilateral to the TCI surgery) from beneath. Each stimulus lasted approximately 1–2 s and the interstimulus interval was approximately 10–15 s. All behavioral tests were conducted by an investigator who was blinded to the previous treatments the animals received.

### 2.4. Micro-CT analysis of bone density of the tibia

Micro-CT analysis was performed on tibias taken from rats on day 8 after TCI using a Bruker scanner (Skyscan 1276) following the manufacturer’s instruction and the methods described previously (Wang et al., 2019; Zhai et al., 2021). In brief, quantification of micro-CT data was calculated for the trabecular bone of proximal tibia from 0.77 to 3.85 mm below the growth plate in the direction of the metaphysis. Bone volume/tissue volume (BV/TV%) was analyzed to evaluate bone density.

### 2.5. Quantitative real-time polymerase chain reaction (qPCR)

The lumbar spinal cord segments (L4-L5) of rats were quickly removed under deep anesthesia and then only the parts of DH of the spinal cord were used for the following experiments. Total RNA was isolated with EasyPure^®^ RNA Kit (TransGen Biotech, ER101-01, Beijing, China) according to the manufacturer’s instructions. cDNA was then synthesized using the TransScript^®^ One-Step gDNA Removal and cDNA Synthesis SuperMix (TransGen Biotech, AT311-03, Beijing, China). The quantitative real-time polymerase chain reaction was performed with the PerfectStart*™* Green qPCR SuperMix (TransGen Biotech, AQ601-04, Beijing, China). The standard conditions were set as follows in QuantStudio 7 Flex: 94°C for 30 s; then 45 cycles at 94°C for 5 s, 55°C for 19 s, and 72°C for 19 s; then 95°C for 15 s, 60°C for 60 s, and a stepwise increase (at 0.05°C/s) to 95°C for 15 s for the melting curve. Primers used for expression analysis were as follows: gene *Tnfa* (TNF-α): forward (5′-3′)- GCATGATCCGAGATGTGGAACTGG; reverse (5′-3′)- CGCCACGAGCAGGAATGAGAAG; gene *Il-1b* (IL-1β): forward (5′-3′)- ATCTCACAGCATCTCGACAAG; reverse (5′-3′)- CACACTAGCAGGTCGTCATCC; gene *Oprm1* (MOR): forward (5′-3′)- ATCCTCTCTTCTGCCATTGGT; reverse (5′-3′)- TGAAGGCGAAGATGAAGACA; and gene *Actb* (β-actin): forward (5′-3′)- CATCCTGCGTCTGGAACCTGG; and reverse (5′-3′)- TAATGTCACGCACGATTTCC. Relative mRNA levels were calculated using the 2^−ΔΔCT^ method. Gene expression was first normalized to the housekeeping control gene *Actb* (β-actin), and then the relative expression of genes of interest was compared with the respective experimental control.

### 2.6. Protein determination

Western blotting analysis was used to qualify temporal changes in protein levels. The spinal cord and DRGs at L4-L5 were quickly removed from deeply anesthetized rats and stored at –80°C. Sequential precipitation procedures were used on the tissue samples which were lysed in ice-cold (4°C) RIPA lysis buffer (Beyotime, P0013B, Shanghai, China) containing a mixture of protease inhibitor (MCE, HY-K0010, NJ, USA), phosphatase inhibitors (MCE, HY-K0021, HYK0022, NJ, USA), and phenylmethylsulfonyl fluoride (Sigma-Aldrich, St. Louis, MO, USA). The total protein was separated by SDS-PAGE and transferred to the PVDF membrane (both from Bio-Rad Laboratories, CA, USA). The following primary antibodies were used: anti-TNF-α (1:1000; Abcam, ab1793, Cambridge, United Kingdom), anti-IL 1β (1:1000; Abcam, ab254360, Cambridge, United Kingdom), anti-MOR (1:2000; Immunostar, 24216, Hudson, USA), anti-HDAC2 (1:1000; Abcam, ab12169, Cambridge, United Kingdom), anti-β-actin (1:1000; CST, 4970, Boston, USA). The membranes were then developed by enhanced chemiluminescence reagents (ChemiSignal*™* Plus, CLINX, Shanghai, China) with horseradish peroxidase-conjugated secondary antibodies (Sangon Biotech, D110087 and D110058, Shanghai, China). Images were acquired with the chemiluminescence instrument (Tanon, Shanghai, China). Data were analyzed with ImageJ. The absolute gray level of each plot is quantified with background subtraction and then normalized with the control plot (β-actin) for comparison.

### 2.7. Immunohistochemistry

Deeply anesthetized rats were perfused transcardially with 0.9% saline followed by 4% paraformaldehyde. The spinal cord and DRGs at L4-L5 segments were removed and postfixed in 4% paraformaldehyde at 4°C for 24 h. After being postfixed, the tissues were transferred into 30% sucrose at 4°C for 3 days for dehydration. The tissues were sectioned at 15 μm thickness for the DH of the spinal cord and 12 μm for the DRG sections. For immunofluorescence staining, sections were blocked in PBS containing 10% goat serum with 0.3% Triton X-100 at room temperature for 2 h and incubated in the primary antibody at 4°C overnight. Sections were then washed in 0.1 M PBS with 0.05% Triton X-100 (pH 7.6) followed by incubating in the secondary antibody at room temperature for 2 h and washing. Sections were mounted on slides and covered with 90% glycerin for observation under a confocal microscope (AR1; Nikon, Tokyo, Japan). The antibodies used included: anti-MOR (1:1000; Abcam, ab134054, Cambridge, United Kingdom), and anti-HDAC2 (1:250; Abcam, ab12169, Cambridge, United Kingdom).

### 2.8. Microbiome DNA sequencing and analysis

Library preparations for DNA sequencing and Illumina NovaSeq sequencing were conducted at Novogene, Inc. (Beijing, China). Fecal DNA was extracted with Magnetic Soil and Stool DNA Kit (TIANGEN, Beijing, China) according to the manufacturer’s instructions. About 10 ng template DNA was subjected to 16SV4 rRNA genes amplification using Phusion^®^ High-Fidelity PCR Master Mix (New England Biolabs). The primer pair used in V4 amplification is 515F (5′-GTGCCAGCMGCCGCGGTAA-3′) and 806R (5′-GGACTACHVGGGTWTCTAAT-3′). Sequencing libraries were generated using TruSeq^®^ DNA PCR-Free Sample Preparation Kit (Illumina, USA) following the manufacturer’s recommendations and index codes were added. The library was validated by Agilent 2100 Bioanalyzer (Agilent Technologies, Palo Alto, CA, USA) and quantified by Qubit 2.0 Fluorometer. At last, the library was sequenced on the Illumina NovaSeq platform according to the manufacturer’s instructions (Illumina, San Diego, CA, USA), and 250 bp paired-end reads were generated. After paired-end reads assembly and quality control, operational taxonomic units (OTUs) were clustered by using Uparse software (Version 7.0.1001), species annotation was performed by using Silva Database based on Mothur algorithm, and phylogenetic relationship was constructed by using MUSCLE software (Version 3.8.31). OTUs abundance information were normalized using a standard of sequence number corresponding to the sample with the least sequences. Differences in microbial communities between groups were evaluated by beta diversity, which was analyzed through binary_jaccard distance metrics calculated by QIIME software (Version 1.9.1) and presented by non-metric multidimensional scaling (NMDS) graph depicted by vegan package in R software (Version 2.15.3).

### 2.9. Propionate and butyrate measurements by gas chromatography/mass spectrometry (GC/MS)

Short-chain fatty acid in feces and serum were extracted and measured with methods similar to that previously described (Zhang S. et al., 2019).

Short-chain fatty acid extraction and derivatization: fresh fecal samples (each 30 mg) were homogenized with 300 μl EDI water (Genie G Water System, Rephile bioscience, Shanghai, China) using the High-throughput tissue grinder (Scientz-48, Ningbo, China) with 3 mm stainless beads (Easybio, BE6638, Beijing, China), followed by incubation at 4°C with shaking for 30 min and centrifugation for 30 min at 13,000 × g to get supernatant. The blood was collected and centrifuged at 5,000 rpm for 15 min at 4°C to get the serum. A total of 100 μl of supernatant (fecal homogenate) or 1:1 diluted serum (in EDI water) was added with 10 μl of 5 M hydrochloric acid to bring the pH to 2, extracted with anhydrous diethyl ether (1:1, v/v) for two times, and dehydrated by adding anhydrous Na_2_SO_4_. The diethyl ether layers (100 μl) were collected together and then derivatized by N, O-Bis (trimethylsilyl)trifluoroacetamide (5 μl; Sigma-Aldrich, 15222-F) at 37°C for 2 h.

Measurements by GC/MS: 7890B gas chromatograph/5977 mass selective detector (Agilent Technologies, Santa Clara, CA, USA) with a HP-5 ms capillary column (30 m × 0.25 mm × 0.25 μm film thickness, Agilent Technologies) was used. The injector, ion source, quadrupole, and the GC/MS interface temperature were, respectively set to 260, 230, 150, and 280°C. The flow rate of helium carrier gas was kept at 1 mL/min, and the solvent delay time was set to 2 min. A total of 1 μl derivatized sample was injected with a split ratio of 10:1. The initial column temperature was 40°C and held for 2 min, ramped to 90°C at the rate of 15°C/min and held for 1 min, and then finally increased to 250°C at the rate of 110°C/min and kept for 3 min. The ionization was carried out in the electron impact (EI) mode at 70 eV. The MS data were acquired in full scan mode from m/z 40 to 400 with an acquisition frequency of 12.8 scans/s to identify specific compounds. The analytes were quantified in the selected ion monitoring (SIM) mode using the target ions (m/z): propionate (75, 131 m/z), butyrate (75, 117, 145 m/z). Data were analyzed with MassHunter programs. The levels of SCFAs were calculated with external standard methods.

### 2.10. Neuro-2a cell culture and drug treatment

Neuro-2a cells (TransGen Biotech, Beijing, China) were used as model systems for cell biology studies. Neuro-2a cells were cultured in complete Dulbecco’s modified Eagle’s medium (DMEM): DMEM (Sigma-Aldrich, St. Louis, MO, USA) supplemented with 10% fetal bovine serum (FBS; Gibco, Los Angeles, CA, USA) and 1% penicillin-streptomycin (Thermo Fisher Scientific, Waltham, MA, USA). The cells were cultured at 37°C in a humidified chamber containing 5% CO_2_ and 95% air. For all the protein determination, neuro-2a cells were cultured at a concentration of 5 × 10^5^ cells/well in 6-well plates to allow them to reach at least 60% confluency before any further treatment (Hou et al., 2017).

Treatment with serum: The blood of animals from each group (Sham, TCI and TCI + LGG group) (*n* = 3) was collected on day 8 after TCI and centrifuged at 5,000 rpm for 15 min at 4°C to get the supernatant (serum). The concentration of collected serum was set as 5%, which replaced half FBS in conventional DMEM complete medium as described above. After treatment for 24 h, total protein of cells was extracted for protein determination.

Treatment with sodium butyrate of concentration gradient: Based on relative literatures (Chang et al., 2014; Jia et al., 2020) and our pilot study, the concentration of sodium butyrate was set as 0.1, 1, and 10 mM, respectively. After treatment for 24 h, total protein of cells was extracted to perform the western blotting assay.

### 2.11. Statistics

All analysis was performed using Prism software (GraphPad, version 9.0.2, San Diego, CA, USA). No statistical methods were used to predetermine sample sizes, but our sample sizes are similar to those reported in previous publications in the field (Hou et al., 2017, 2018; Ma et al., 2022). The subgroups were randomly selected and all detections were done by experimenters who were blind to the protocol. Results from assessment of mechanical allodynia over time were analyzed using two-way ANOVA with repeated measurements followed by multiple comparisons. Alterations of expression of the proteins, bone intensity, abundance of microbiota, and SCFAs among three groups were tested with one-way ANOVA with repeated measures followed by Dunnett’s multiple comparison tests. For other measurements between 2 groups, two-tailed, unpaired Student’s *t*-test was used. All data were expressed as mean ± SEM.

## 3. Results

### 3.1. LGG supplementation alleviates TCI-induced mechanical allodynia, spinal inflammation and the bone destruction

We began by examining alterations of mechanical allodynia induced by TCI in rats with or without LGG supplementation. Continuous LGG supplementation significantly delayed the onset of mechanical allodynia for about 4 days, nearly abolished mechanical allodynia during day 3 to day 5, and reduced mechanical allodynia by approximately 30–50% during day 7 to day 11. LGG supplementation did not change mechanical allodynia after 11 days until the last examination on day 21, although LGG was continuously supplemented. In contrast, LGG supplementation didn’t produce any effect on the mechanical sensitivity of the hind paw in control animals (Figure 1A).

Inflammation is associated with chronic pain. We thus examined the proinflammatory cytokines TNF-α and IL-1β in the DH to evaluate the possible effect of LGG supplementation on inflammation on day 8 after TCI when LGG treatment produced the significant and stable analgesic effect on BCP. We found that TCI-induced increased mRNA level and protein expression of TNF-α and IL-1β were significantly suppressed by LGG treatment (Figures 1B, C). Tumor-induced osteolytic bone destruction was demonstrated to contribute to the development of BCP (Zajączkowska et al., 2019). The result of trabecular bone density in the tibia showed that TCI-induced bone destruction was also significantly improved following LGG treatment on day 8 after LGG (Figure 1D). We did not examine the possible alterations of inflammation and bone density after day 11 when LGG did not change painful sensitivity in TCI animals. These findings indicate that LGG supplementation can inhibit TCI-induced production of BCP and the associated spinal inflammation and alleviate the bone destruction in the early stage following LGG treatment.

### 3.2. LGG supplementation increases expression of MOR in the DH and enhances morphine analgesia

**FIGURE 2:**
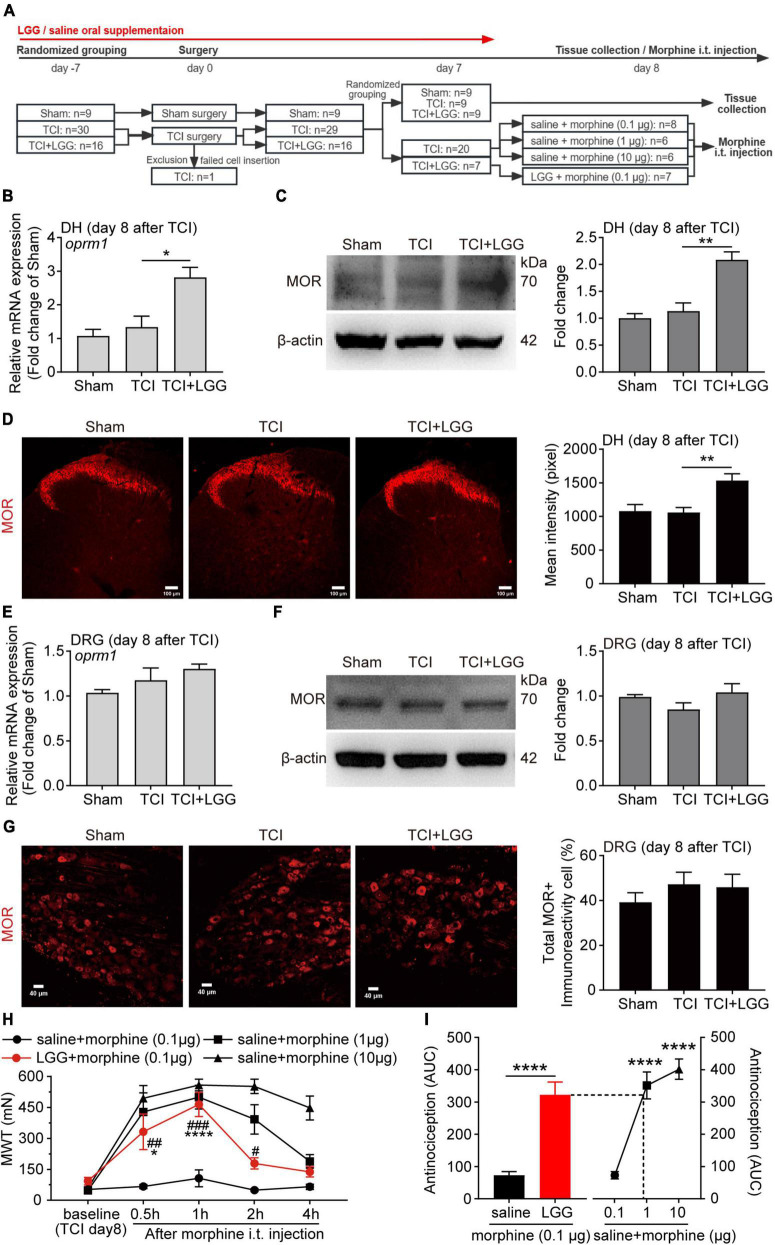
Lactobacillus rhamnosus GG (LGG) supplementation increases expression of MOR in the spinal dorsal horn (DH) and enhances morphine analgesia on day 8 after tumor cell implantation (TCI). **(A)** Schematic illustration of the experimental process. **(B,C)** Expression of MOR mRNA **(B)** and protein **(C)** in the spinal DH. *n* = 3 in each group. One-way ANOVA with Dunnett’s multiple comparison test, *p < 0.05, **p < 0.01. MOR, μ-opioid receptor. **(D)** Immunofluorescence showing expression of MOR in the spinal DH (left). Histogram showing the mean intensity of MOR immunofluorescent activity (right). A total of six slices from three animals in Sham, eleven slices from three animals in other groups. One-way ANOVA with Dunnett’s multiple comparison test, **p < 0.01. Scale bar = 100 μm. **(E,F)** Expression of MOR mRNA **(E)** and protein **(F)** in the L4-L5 dorsal root ganglion (DRG). **(G)** Immunofluorescence showing expression of MOR in the L4-L5 DRG (left). Histogram showing the ratio of total MOR-positive neurons in the DRG (right). A total of eight slices from three animals in each group. Scale bar = 40 μm. **(H)** MWT after morphine intrathecal (i.t.) injection in rats treated with LGG or saline on day 8 after TCI. *n* = 8 in saline + morphine (0.1 μg), *n* = 7 in LGG + morphine (0.1 μg), *n* = 6 in other groups. Two-way ANOVA with Dunnett’s multiple comparison test, *p < 0.05, ****p < 0.0001 LGG + morphine 0.1 μg vs. LGG + morphine 0.1 μg baseline; Two-way ANOVA with Tukey’s multiple comparison test, #*p* < 0.05, ##*p* < 0.01, ###*p* < 0.001 LGG + morphine 0.1 μg vs. saline + morphine 0.1 μg. MWT, mechanical withdrawal threshold. **(I)** The area under MWT-time curve (AUC) in **(H)**, from the baseline (TCI day8) to 1h after i.t. injection. *n* = 8 in saline + morphine (0.1 μg), *n* = 7 in LGG + morphine (0.1 μg), *n* = 6 in other groups. Unpaired Student’s *t*-test (left), ****p < 0.0001. One-way ANOVA with Tukey’s multiple comparison test, ****p < 0.0001 vs. saline + morphine (0.1 μg).

The endogenous opioid system is critical to the pain process. Studies have shown that expression of MOR in the DRG and the DH is suppressed in rodents with BCP (Yamamoto et al., 2008; Zhu et al., 2017) and could be activated by some specific Lactobacillus strains *in vitro* (Rousseaux et al., 2007). To understand the possible mechanism that may underlie the analgesic effect of LGG supplementation on TCI-induced pain, we examined expression of MOR in the DRG and the DH following *in vivo* LGG treatment by western blot and immunofluorescent staining. The tissues were collected on day 8 after TCI when LGG treatment produced the significant and stable analgesic effect on BCP (see Figures 1A, 2A). The level of mRNA and the protein expression of MOR was not significantly changed by TCI treatment. Interestingly, LGG treatment significantly increased levels of mRNA and the protein expression of MOR in the DH after TCI (Figures 2B–D). However, in the DRG, the expression of mRNA and protein of MOR was not changed following the same treatment at the same time (Figures 2E–G). These findings suggest that the increased expression of MOR in the DH may be a potential mechanism for analgesia following the oral LGG supplementation.

Given that LGG treatment induced an increased expression of MOR in the DH, we continued to test whether LGG supplementation could facilitate the analgesic effect of morphine, which is an agonist of MOR and a drug used widely in patients bearing with cancer pain. We first examined the analgesic effects of intrathecal administration of morphine at different doses (0.1, 1, and 10 μg) in rats with mechanical allodynia on day 8 after TCI. The intrathecal injection of morphine produced a dose-related analgesia. Morphine treatment at 0.1 μg had limited analgesic effect on the mechanical allodynia (Figure 2H). We then tested effects of combination of morphine (0.1 μg) and the LGG continuous supplementation (continuously supplied from 7 days before TCI to the day of this test) on the mechanical allodynia in TCI animals. The LGG supplementation significantly boosted the inhibitory effect of morphine (0.1 μg) on the allodynia. The inhibition began within 0.5 h, reached peak at 1 h, and lasted for at least 2 h. The inhibition amplitude reached the similar level of inhibition of morphine at 1 μg (Figure 2H). We further analyzed the synergistic analgesic effect of the combination of LGG and morphine on BCP and compared it with morphine treatment alone by calculating the area under curve (AUC) in Figure 2H, from the baseline (TCI day8) to 1h after i.t. injection, as shown in Figure 2I. The analgesic effect of the combination of morphine at 0.1 μg with LGG treatment could reach the level of analgesia produced by morphine alone at 1 μg. Taken together, these results demonstrate that continuous oral supplementation of LGG can greatly facilitate morphine analgesia and produce the synergistic analgesic effect of morphine on the cancer pain.

### 3.3. LGG supplementation restores specific microbiota and increases microbial metabolite butyrate in the feces and serum

**FIGURE 3:**
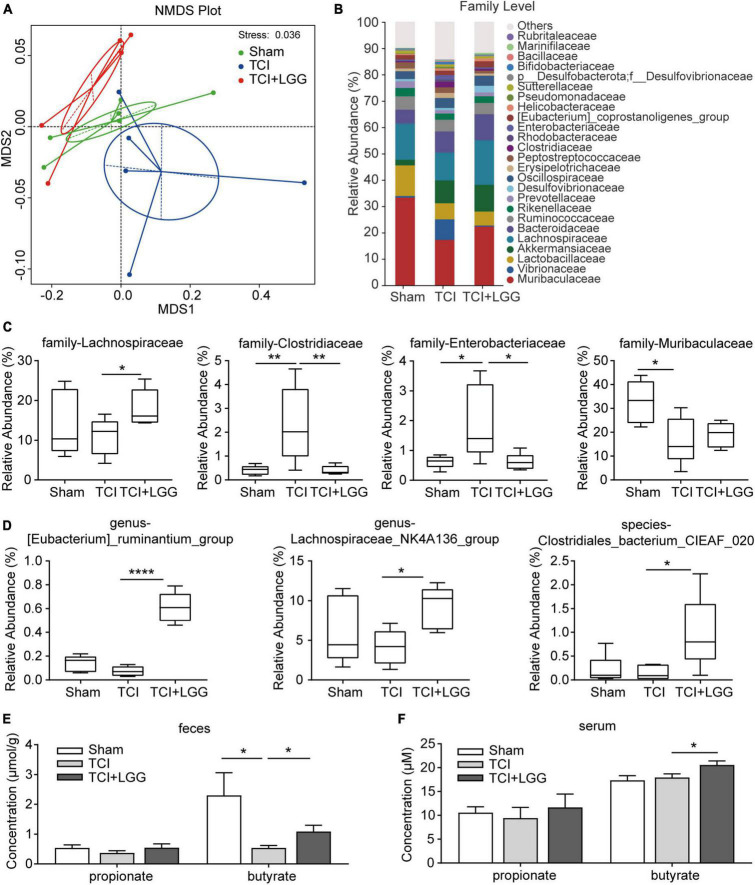
Restored specific microbiota abundance and increased butyrate in the feces and serum on day 8 after tumor cell implantation (TCI) following Lactobacillus rhamnosus GG (LGG) supplementation. **(A)** Non-metric multi-dimensional scaling (NMDS) analysis based at Binary_Jaccard metrics showing the major differences in the composition of microbiota. n = 6 in Sham, n = 5 in TCI and TCI + LGG. **(B)** Stacked bar-chart showing gut microbiota composition at the family level. n = 6 in Sham, n = 5 in TCI and TCI + LGG. **(C)** Relative abundance of the major microbiota families from **(B)** that was significantly changed following LGG supplementation in TCI-rats. n = 6 in Sham, n = 5 in TCI and TCI + LGG. One-way ANOVA with Dunnett’s multiple comparison test, *p < 0.05, **p < 0.01. **(D)** Relative abundance of specific gut microbiota that positively correlated with short-chain fatty acids (SCFAs) production. n = 6 in Sham, n = 5 in TCI and TCI + LGG. One-way ANOVA with Dunnett’s multiple comparison test, *p < 0.05 ****p < 0.0001. **(E,F)** Propionate and butyrate concentrations in the feces **(E)** and serum **(F)** detected by gas chromatography/mass spectrometry (GC/MS). Butyrate in feces: n = 5 in TCI + LGG; butyrate in serum: n = 5 in Sham; n = 6 in other groups. One-way ANOVA with Dunnett’s multiple comparison test, *p < 0.05.

The dysbiosis of gut microbiota and decreased Lactobacillus was reported in patients with cancer pain (Zhang et al., 2022). We hypothesized that the microbiota and metabolite might be regulated by LGG supplementation and thus led to pain relief and the biochemical changes as described in last paragraph. To verify this hypothesis, we first performed 16S rDNA sequencing analysis with feces in the colon collected from rats at day 8 in each of the groups with treatment of sham surgery, TCI, or TCI + LGG. The NMDS analysis showed that the microbial composition in the TCI-rats was distinct from the sham control. With LGG supplementation, the clustering of samples was located closer to the rats with sham surgery, but distantly from the TCI-treated samples (Figure 3A), suggesting that LGG supplementation possibly restored the alterations of gut microbial diversity after TCI treatment. Similarly, the relative abundance of the top 25 gut microbiota at the family level also indicated that LGG supplementation remodeled microbiota structure, which was previously altered by TCI treatment (Figure 3B). Among these microbiota families, Lachnospiraceae, which was recognized as a SCFAs producer (Zhang J. et al., 2019; Hiraishi et al., 2022) and reported to inhibit inflammation and cancer (Vacca et al., 2020), was significantly increased in the colon of TCI-rats following LGG supplementation. Clostridiaceae (Muñiz Pedrogo et al., 2019) and Enterobacteriaceae (Ganten et al., 2006; Hiraishi et al., 2022), both were associated with inflammation in the colon and described as opportunistic gut pathogens, were significantly increased in the feces after TCI treatment and reversed by LGG supplementation. Muribaculaceae, which exhibited metabolic functions like glycan degradation (Lagkouvardos et al., 2019), was significantly reduced in the colon after TCI treatment, but not reversed by LGG here (Figure 3C).

Short-chain fatty acids play a critical role in the microbiota-gut-brain axis (Dalile et al., 2019) and the regulation of neuropathic pain (Guo et al., 2019). We continued to examine whether the LGG-restored colon microbiota could affect SCFAs levels in the feces and serum of TCI-rats. We first screened the 16s rDNA sequencing results by *t*-test (*p* < 0.05) to find specific bacteria that were significantly changed following LGG supplementation in TCI-rats. We found that, among the bacteria in different levels, several specific SCFA-related bacteria were significantly increased by LGG supplementation, they were (Eubacterium)_ruminantium_group genus (Balows, 1992; Korpela et al., 2014), Lachnospiraceae_NK4A136_group genus (Hippe et al., 2011; Zhang J. et al., 2019; Stadlbauer et al., 2020), and Clostridiales_bacterium_CIEAF_020 species (Xu et al., 2021; Figure 3D). By further GC/MS analysis, dramatic decrease of butyrate but not propionate was detected in feces of TCI-treated rats, with increased butyrate detected both in feces and serum of TCI-rats with LGG supplementation (Figures 3E, F). These findings indicate that LGG supplementation remodels the composition of gut microbiota and metabolites, particularly the butyrate in SCFAs.

### 3.4. Butyrate supplementation inhibits BCP and increases expression of MOR in the DH

**FIGURE 4:**
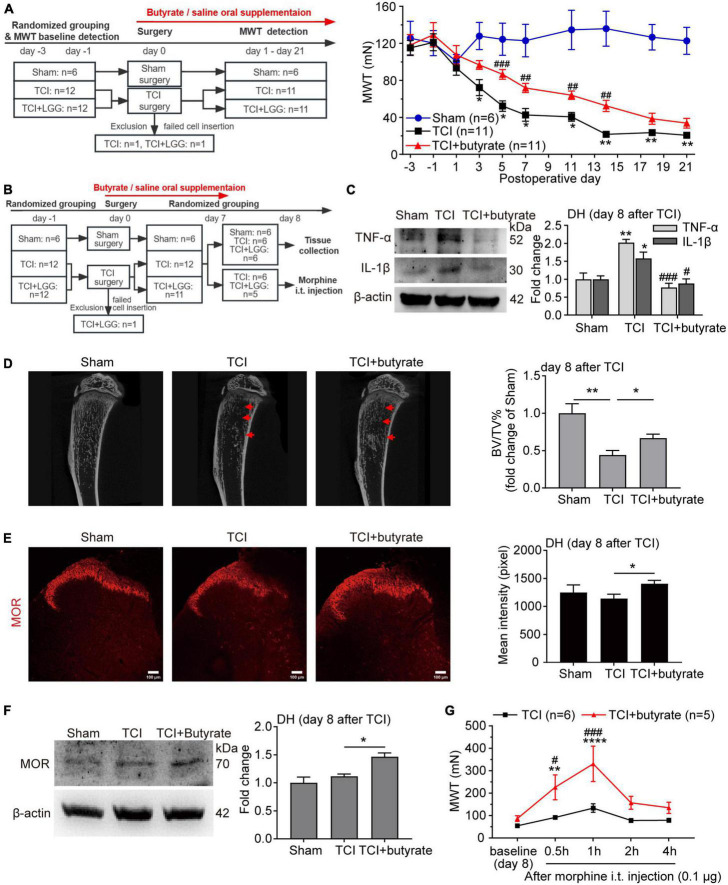
Butyrate supplementation inhibits bone cancer pain, increases expression of μ-opioid receptor (MOR) in the dorsal horn (DH), and enhances morphine analgesia. **(A)** Reduced mechanical allodynia manifested as increased MWT following the oral supplementation of butyrate (100 mg/kg/day) in rats with tumor cell implantation (TCI). Two-way ANOVA with Tukey’s multiple comparisons test *p < 0.05, **p < 0.01 TCI vs. Sham; ##*p* < 0.01, ###*p* < 0.001 TCI + butyrate vs. TCI. **(B)** Schematic illustration of the experimental process of **(C–G)**. **(C)** Expression of tumor necrosis factor alpha (TNF-α) and interleukin-1beta (IL-1β) protein in the spinal DH on day 8 after TCI. *n* = 3 in each group. One-way ANOVA with Dunnett’s multiple comparison test, *p < 0.05, **p < 0.01 TCI vs. Sham; #*p* < 0.05, ###*p* < 0.001 TCI + butyrate vs. TCI. **(D)** Representative micro-CT images (left) showing bone microstructure, and quantification (right) showing BV/TV in the proximal part of tibia trabecular bone from rats on day 8 after TCI. *n* = 6 in Sham, *n* = 5 in other groups. One-way ANOVA with Dunnett’s multiple comparison test, *p < 0.05, **p < 0.01. BV/TV, bone volume/tissue volume. **(E)** Immunofluorescence showing expression of MOR in the spinal DH (left) on day 8 after TCI. Histogram showing the mean intensity of MOR immunofluorescent activity (right). A total of seven slices from three animals in Sham, eleven slices from three animals in TCI, twelve slices from three animals in TCI + butyrate. One-way ANOVA with Dunnett’s multiple comparison test, *p < 0.05. Scale bar = 100 μm. **(F)** Expression of MOR protein in the spinal DH on day 8 after TCI. *n* = 3 in each group. One-way ANOVA with Dunnett’s multiple comparison test, *p < 0.05. **(G)** MWT after morphine intrathecal (i.t.) injection (0.1 μg) in rats treated with butyrate or saline on day 8 after TCI. *n* = 6 in TCI, *n* = 5 in TCI + butyrate. Two-way ANOVA with Dunnett’s multiple comparisons test **p < 0.01, ****p < 0.0001 TCI + butyrate vs. TCI + butyrate baseline (day 8); Two-way ANOVA with Šídák’s multiple comparisons test, #*p* < 0.05, ###*p* < 0.001 TCI + butyrate vs. TCI. MWT, mechanical withdrawal threshold.

Given that LGG supplementation remodels the composition of gut microbiota and metabolites, particularly the butyrate in SCFAs, we further examined whether butyrate would be a key mediator, by which LGG inhibited the development of BCP and increased the expression of MOR. Continuous butyrate supplementation significantly alleviated TCI-induced mechanical allodynia (Figure 4A) in the pattern similar to LGG (also see Figure 1A). We collected tissues on day 8 after TCI, which was 8 days after continuous butyrate treatment (Figure 4B), and found that butyrate inhibited the expression of TNF-α and IL-β in the DH (Figure 4C) and reduced tibial bone destruction (Figure 4D). Such a continuous butyrate treatment also significantly increased expression of MOR in the DH (Figures 4E, F) and enhanced the analgesic effect of morphine (0.1 μg, i.t., in 10 μl) (Figure 4G). These findings indicate that butyrate produces an analgesic effect on TCI-induced pain that is quite similar to LGG, suggesting that the increased butyrate in the colon and serum during LGG supplementation may be potentially a critical mediator for LGG to increase MOR expression and alleviate pain.

### 3.5. The LGG and butyrate supplementation inhibited expression of HDAC2 and increased expression of MOR in the DH

**FIGURE 5:**
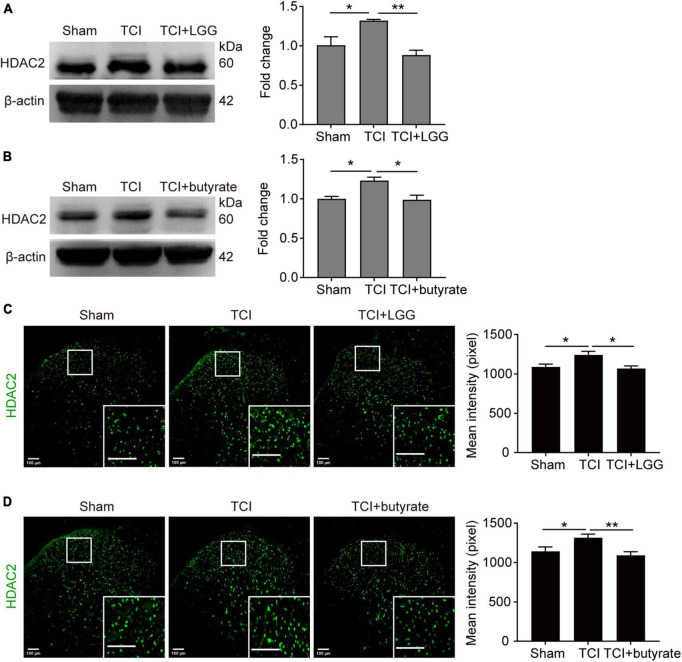
Suppressed expression of HDAC2 in the spinal dorsal horn (DH) on day 8 after tumor cell implantation (TCI) following Lactobacillus rhamnosus GG (LGG) or butyrate supplementation. **(A,B)** Expression of HDAC2 protein in the spinal DH after LGG **(A)** or butyrate **(B)** supplementation. *n* = 3 in each group. One-way ANOVA with Dunnett’s multiple comparison test, *p < 0.05, **p < 0.01. HDAC2, histone deacetylase 2. **(C,D)** Immunofluorescence showing expression of HDAC2 (green) in the spinal DH after LGG **(C)** or butyrate **(D)** supplementation. Histogram showing the mean intensity of HDAC2 immunofluorescent activity. A total of six slices from three animals in Sham, eight slices from three animals in TCI, seven slices from three animals in TCI + LGG in **(C)**. A total of six slices from three animals in Sham, seven slices from three animals in other groups in **(D)**. One-way ANOVA with Dunnett’s multiple comparison test, *p < 0.05, **p < 0.01. Scale bar = 100 μm.

**FIGURE 6:**
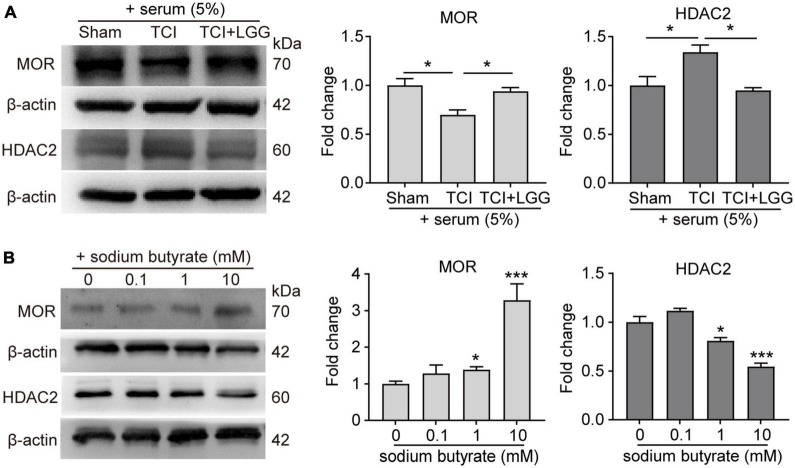
Expression of increased μ-opioid receptor (MOR) and decreased histone deacetylase 2 (HDAC2) protein in Neuro2a cells following treatment of rat serum and sodium butyrate. **(A)** Expression of MOR and HDAC2 protein in Neuro2a cells after incubation for 24 h with serum (5%) collected from rats on day 8 after surgery in the Sham, TCI and TCI + LGG group. *n* = 3 independent experiments in each group. One-way ANOVA with Dunnett’s multiple comparison test, *p < 0.05. **(B)** Expression of MOR and HDAC2 protein in Neuro2a cells after incubation for 24 h with 0.1, 1, and 10 mM sodium butyrate. *n* = 3 independent experiments in each group. One-way ANOVA with Dunnett’s multiple comparison test, *p < 0.05 ***p < 0.001 vs. 0 mM.

Studies have reported that butyrate and its derivatives may promote the transcription and expression of MOR through inhibition of HDACs (Hwang et al., 2007, 2010; Kukkar et al., 2014; Hou et al., 2017), and that suppression of increased HDAC2 in the spinal cord alleviates mechanical allodynia in rats with BCP (He et al., 2020). We examined effects of administration of LGG and butyrate, respectively on expression of MOR and HDAC2 in the DH. The LGG and butyrate were administrated separately *in vivo* and *in vitro*, respectively. Our western blot analysis and immunofluorescence staining showed that *in vivo* treatment of LGG or butyrate inhibited TCI-induced increased expression of HDAC2 in the DH (Figures 5A–D). Our *in vitro* experiments in neuro-2a cells showed that the expression of MOR was increased and the expression of HDAC2 was decreased, following 24 h incubation of the neuro-2a cells with 5% serum from TCI-rats previously treated with LGG (Figure 6A). The induction of MOR and inhibition of HDAC2 were simultaneously detected in neuro-2a cells following 24 h incubation with sodium butyrate (0.1, 1, and 10 mM) (Figure 6B). These results provide further evidence that HDAC2 may be a potential downstream target for LGG and butyrate supplementation to induce MOR expression in the DH.

## 4. Discussion

This study reveals that oral supplementation of LGG can inhibit BCP in TCI rats. The analgesic effect may be mediated by butyrate through increasing the expression of MOR in the DH. The main findings include 4-fold. (i) LGG significantly inhibited the onset of the mechanical allodynia for 3–4 days and reduced the ongoing pain for up to 11–14 days; (ii) LGG increased the expression of MOR in the DH and synergistically enhanced the analgesic effect of morphine; (iii) LGG restored the gut microbiota composition and increased butyrate in the feces and serum, and oral administration of butyrate produced similar effects to that of LGG on the mechanical allodynia and MOR expression; (iv) HDAC2 may serve as the target of butyrate and LGG to promote the expression of MOR. This study provides new evidence supporting the idea that LGG can inhibit BCP through butyrate by increasing MOR in the DH. LGG has the potential for modulating gut microbiota and may serve as a treatment strategy for cancer pain management in clinic.

Lactobacillus rhamnosus GG delays the onset of mechanical allodynia and reduces ongoing pain in the early stage after TCI treatment. This analgesic effect of LGG may be realized through suppressing the proinflammatory cytokines TNF-α and IL-1β and elevating MOR expression in the DH. LGG also produces great synergistic analgesic effects on BCP when it is used with morphine. These effects of LGG may be mediated through butyrate since the LGG supplementation in TCI rats results in an increased butyrate in feces and serum and that butyrate can produce similar effects on mechanical allodynia, the inflammatory cytokines and MOR as LGG does. Meanwhile, our results showed that both LGG and butyrate supplementation suppressed TCI-induced trabecular bone loss. It was reported that butyrate could stimulate bone formation (Tyagi et al., 2018). Thus, LGG treatment-induced increased butyrate may contribute to the reduced bone destruction following LGG supplementation.

Studies have shown that butyrate is a pan-inhibitor of class I and class II HDACs (Candido et al., 1978; Arpaia et al., 2013). Inhibiting HDAC2 can alleviate cancer mechanical allodynia (Hou et al., 2017, 2018). Thus, butyrate may suppress BCP through the inhibition of HDAC2. Studies also suggested that expression of MOR could be inhibited by HDACs (Kim et al., 2004; Hwang et al., 2010; Zhu et al., 2017). Here, we found that increased expression of HDAC2 in the DH of TCI rats was inhibited following LGG or butyrate treatment. In neura-2a cells, we found that expression of HDAC2 was inhibited after incubation with serum from rats with TCI + LGG treatment, or sodium butyrate solution. These findings indicate a correlation among the butyrate, HDAC2, and MOR, suggesting that HDAC2 may be the target of butyrate and LGG to modulate MOR and alleviate mechanical allodynia in TCI rats.

Butyrate has also been reported to mediate the inhibition of inflammation and modulate the immune system in diseases such as inflammatory bowel disease, diabetes, cardiovascular diseases, and pain disorders (Russo et al., 2018). It was suggested that butyrate could suppress the inflammatory process through regulating cytokine expressions including IL-6, TNF-α, and IL-10 in regulatory T-cells, macrophages, and/or neutrophils in the peripheral system, especially in the intestine (Arpaia et al., 2013; Chang et al., 2014). In the nervous system, butyrate was found to inhibit TNF-α in the spinal cord and sciatic nerves of rodents in the neuropathic pain model (Kukkar et al., 2014; Russo et al., 2016). Since butyrate can suppress and inactivate HDACs and the suppression of HDACs by another pan-inhibitor trichostatin A inhibits TNF-α (Vinolo et al., 2011), HDACs suppression may be one of the targeting pathways for butyrate to inhibit expression of TNF-α in the central nervous system. It was also suggested that butyrate might inhibit IL-1β through the inactivation of NF-κB pathway (Jia et al., 2020), but it remains unknown whether butyrate inhibits IL-1β by suppressing HDACs. Here we show that butyrate inhibits the expression of TNF-α and IL-1β in the DH and HDAC2 at the early stage of BCP. Taken together, these findings indicate that butyrate may inhibit cytokines through an HDAC2-dependent pathway.

Another interesting finding in our study is that expression of MOR was significantly increased in the DH following LGG supplementation. In the DH, MOR is mainly located in the laminae I-II and is distributed in the axon terminals of DRG neurons as well as some projection neurons and interneurons (Corder et al., 2018). The MOR located at the axon terminals of DRG neurons are synthesized from the soma of neurons at DRG. Our result shows that LGG increased expression of MOR only in the superficial layers of the DH, but not in the DRG neurons. This increased expression of MOR in the DH may underlie the analgesic effect of LGG.

There are several limitations in this study, for instance, whether and how HDAC2 inhibits MOR; how LGG and butyrate inhibits expression of HDAC2. In addition, we used only mechanical allodynia as a sign of BCP and ignored the other symptoms such as the spontaneous pain and the evoked thermal hyperalgesia indicated in TCI-rats in previous studies (Liu et al., 2013; Wang et al., 2020). Further studies are also required to elucidate why analgesic effects of continuous treatment of LGG and butyrate lasted only for less than 2 weeks.

In conclusion, this study reveals the analgesic effects of LGG and butyrate on mechanical allodynia in BCP and the synergistic analgesic effect of LGG and butyrate when used in combination with morphine for BCP. The butyrate-HDAC2-MOR pathway may be the underlying mechanism for the analgesic effect of LGG.

## Data availability statement

The datasets presented in this study can be found in online repositories. The names of the repository and accession number can be found below: https://www.ncbi.nlm.nih.gov/, RJNA953343.

## Ethics statement

The animal study was reviewed and approved by Southern University of Science and Technology Animal Care and Use Committee.

## Author contributions

JX, WY, and X-JS designed research studies. WY, JX, HL, ZX, YZ, and CL conducted experiments and acquired data. WY, JX, X-JS, and KZ analyzed data. WY and X-JS wrote the manuscript. All authors read and approved the final manuscript.
